# A Decade of Urology Residency Match Trends: Growth, Competition, and Diversity

**DOI:** 10.7759/cureus.84674

**Published:** 2025-05-23

**Authors:** Abdul-Jawad J Majeed, Dennis Head, Jay D Raman

**Affiliations:** 1 Urology, Penn State College of Medicine, Hershey, USA

**Keywords:** applicant demographics, diversity in medicine, graduate medical education, urology match, urology residency

## Abstract

Introduction

Urology residency positions in the United States are filled through an early, independent match process administered by the American Urological Association (AUA). As a highly competitive specialty, understanding long-term trends in matching outcomes and applicant demographics is essential for workforce planning and equity. This study analyzed trends in match rates, applicant characteristics, and training capacity from 2015 to 2024.

Methods

Publicly available AUA match data from 2015 to 2024 were analyzed. Variables included applicant counts, residency positions, match rates, medical training status, sex, and race/ethnicity for 2024. Statistical analyses included linear regression, correlation, Chi-square tests, and ANOVA to assess trends and subgroup differences.

Results

Residency positions increased by 33% over the study period (296 in 2015 to 394 in 2024, +11 per year, p<0.001), with applicants rising from 433 to 500 (+13 per year, p=0.01). The overall match rate averaged 75% (±5.7%) and remained stable over time. First-time medical seniors had significantly higher match rates (81%) than previous graduates (59%) and international graduates (33%) (p<0.001). The number of female applicants nearly doubled, comprising 40.6% of the 2024 pool, with match rates exceeding those of males in recent cycles. In 2024, Black and Hispanic applicants had lower match rates compared to their White and Asian peers.

Conclusion

The urology match has grown but remains highly competitive. While gender diversity has improved, racial and ethnic disparities have persisted. Continued expansion of residency positions and targeted diversity initiatives are essential to address workforce shortages and promote equity in urological training.

## Introduction

Unlike most specialties in the National Resident Matching Program (NRMP), urology conducts an earlier, independent match process facilitated by the American Urological Association (AUA) and Society of Academic Urologists (SAU). This match is highly competitive, with an average match rate of approximately 71% in recent years, leaving a proportion of applicants unmatched [1,2]. The match outcomes fluctuate based on the size of the applicant pool and the number of available positions. For example, the match rate peaked at 85% in 2019 but fell to 65.6% in 2022 amid a surge in applicants [2,3]. These trends likely influence applicant behavior, as students weigh their odds of matching when choosing specialties.

Tracking trends in the urology match is important for several reasons. First, match outcomes serve as a barometer for the future of the urologist workforce. The U.S. faces a growing need for urologists due to an aging population and retirements within the specialty [4]. Although the number of residency positions has gradually increased, it remains uncertain whether growth is keeping pace with demand. Second, match data can highlight progress or gaps in diversity within urology, which is historically one of the least diverse fields. Women comprise only 11.8% of practicing urologists, while black physicians comprise only 2% [5,6]. Efforts such as mentorship programs, scholarships, and initiatives by organizations such as the Society of Women in Urology and the R. Frank Jones Urologic Society aim to expand this pipeline. Analyzing match outcomes helps assess the impact of these initiatives. Finally, studying match trends provides valuable guidance for medical students and educators. Understanding patterns, particularly those affecting reapplicants or international medical graduates, can help tailor advice and empower students to make informed, strategic decisions.

Our study builds on the existing literature by examining a decade of AUA-SAU matching data from 2015 to 2024 in detail. We aimed to characterize trends in match rates, the expanding applicant and program pool, and demographic changes, and discuss their implications for the urology workforce and future applicants.

## Materials and methods

Data sources

Publicly available data on urology resident match outcomes from 2015 to 2024 were sourced from American Urological Association (AUA) match reports [1,7]. Data included applicant and match counts by training status and demographics. A total of 4,549 records were scanned. All data were de-identified and aggregated, and no individual-level data were available. Military urology positions, which use a separate match process, were excluded but represented a small fraction of the total positions [8]. Applicant race/ethnicity data were only available beginning with the 2024 match; therefore, the race-based analysis was limited to that year. The terms used in this paper for identifying race/ethnicity are as available in the National Residency Match Program (NRMP) database. These terms reflect self-identified selections by the participants. 

Study variables

Each year, we recorded the number of registered applicants, available positions, and matched versus unmatched applicants. Applicants were categorized according to their medical training status as follows: 1. First-time senior applicants were defined as U.S. or Canadian Doctor of Medicine (MD)/Doctor of Osteopathic Medicine (DO) students in their final year, 2. Previous graduates included reapplicants and those who had graduated prior to the match year. 3. International medical graduates (IMGs), both U.S. citizens and foreign nationals, were grouped as “international”. Non-binary/undisclosed entries in 2024 were excluded because of their low counts. Key outcomes included the annual match rates by group, total applicants, and available positions.

Data analysis

Data were entered into Microsoft Excel and analyzed using R software (R Foundation for Statistical Computing, Vienna, Austria). Trends were assessed using linear regression with year as the independent variable. We estimated annual changes (slopes) and used p-values to determine statistical significance. Group comparisons (e.g., match rates by training status or sex) were evaluated using Chi-square tests for categorical data and ANOVA or t-tests. All statistical tests were two-tailed, with significance set at p < 0.05.

Inclusion/exclusion criteria

All applicants who submitted a rank list from 2015 to 2024 were included in this study. Only primary match data were analyzed, and post-match processes were excluded. As all data were publicly available and de-identified, the study was Institutional Review Board (IRB) exempt.

## Results

Growth of applicants and positions

Over the past decade, both the number of urology residency positions and the number of applicants have increased substantially. Residency slots grew from 296 in 2015 to 394 in 2024 (+33%).7,9 The expansion in positions was steady, averaging roughly +11 additional spots per year (p < 0.001). The applicant pool also expanded, albeit at a slightly lower rate. Applicants submitting rank lists rose from 433 (2015) to 500 (2024) [7,9]. This increase in applicants averaged approximately +13 per year (p = 0.01).

Overall match rates

**Figure 1:**
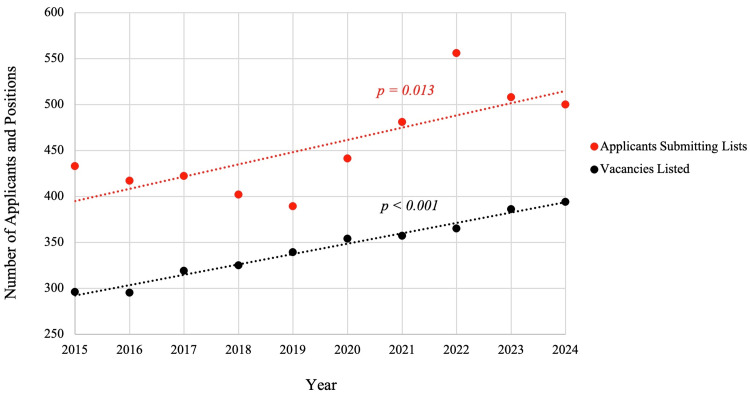
Trends in residency applicants vs. available positions from 2015 to 2024

The chance of matching for a given applicant fluctuated from year to year, with an overall mean match rate of approximately 75% (3,393 of 4,549 applicants, ±5.7% SD) from 2015 to 2024. Match rates ranged from 66% (n = 365 of 556 in 2022) to 85% (n = 330 of 389 in 2019). By contrast, the highest match rate was in 2019, when 85% of applicants matched​, a year notable for a slowdown in applicant growth alongside continued program expansion. By 2024, the match rate was 77%​ (n = 385 of 500), mostly above the decade average. Trendlines suggest that the expansion of positions kept pace with applicants (Figure 1).

Applicant type and match success

**Figure 2:**
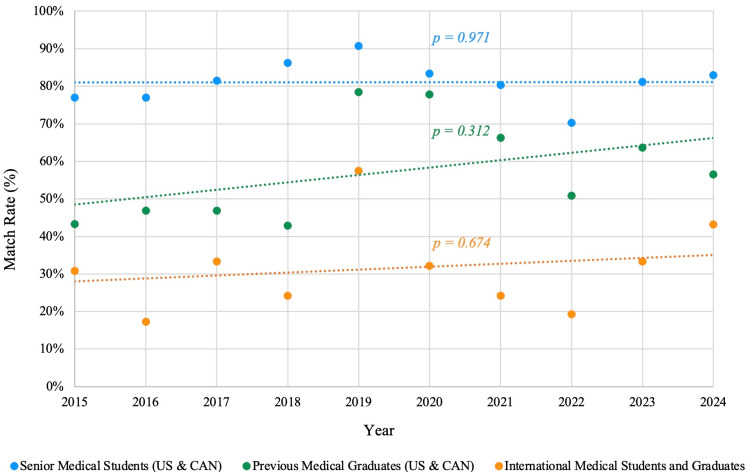
Match rates by training status from 2015 to 2024

The match outcomes differed markedly according to the applicant training status (Figure 2). First-time senior applicants had a much higher success rate than the other applicants. From 2015 to 2024, first-time senior applicants had an average match rate of about 81% (n = 2,987/3,708), compared to about 59% (n = 295/503) for previous graduates (reapplicants), and approximately 33% (n = 111/338) for international medical graduates. This difference was statistically significant (p < 0.001 for both comparisons). In 2024, the match rates were 83% (n = 340/410) for first-time seniors, 57% (n = 26/46) for reapplicants, and 43% (n = 26/60) for IMGs. However, the data suggest that this gap may have been slightly narrowed. The IMG match rates rose from approximately 27% (n = 36/134) during 2015 to 2020 to 43% (n = 26/60) in 2024. Similarly, previous medical graduate applicant match rates improved from 43% (n = 19/44) to 57% (n = 26/46). Nonetheless, at least 40-50% of reapplicants and the majority of IMG applicants failed to match.

Gender trends

**Figure 3:**
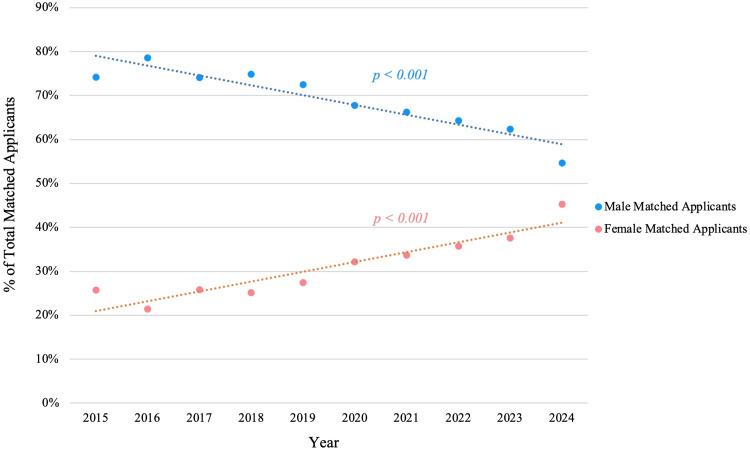
Gender composition of matched urology applicants (2015 to 2024) Gender composition of matched urology applicants from 2015 to 2024. The proportion of female matched applicants increased significantly over time (β = 2.24% per year, p < 0.001), while the proportion of male matched applicants decreased (β = –2.24% per year, p < 0.001).

The gender composition of urology applicants and matriculating residents has changed noticeably from 2015 to 2024 (Figure 3). On average, male applicants outnumber female applicants each year. The mean numbers of male and female applicants were 313 and 134, respectively, with a roughly 70:30 ratio (p<0.001). In 2015, for instance, out of 433 applicants, 112 were women (25.9%)​. By 2024, women comprised approximately 40.6% of the applicant pool (n = 203/500). This trend of increasing female participation was strongly positive over time (r = 0.89, p<0.001). The number of female applicants roughly doubled over the decade, whereas the number of male applicants increased only slightly. As a result, the gender gap narrowed from roughly three male applicants for every female in 2015 to about 1.5:1 in 2024.

**Figure 4:**
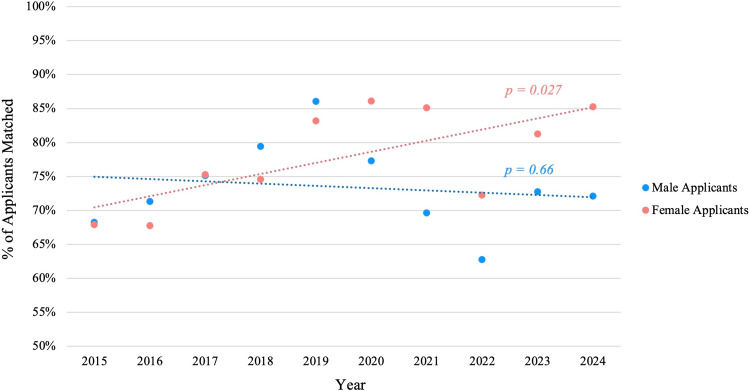
Match rates by gender among urology applicants, 2015 to 2024 Female match rates increased significantly over time (p = 0.027), while male match rates showed no significant change (p = 0.66).

Across the full 10-year span, female and male applicants had roughly equivalent overall success. The mean match rate for female applicants was 78.5% (n = 1055/1343) versus 72.9% (n = 2279/3126) for males, a difference that was not statistically significant (p = 0.168). However, this aggregate view masks recent divergence. Female match rates trended upward significantly in the latter part of the decade (r = 0.69, p = 0.03), and in the most recent years, women actually matched at higher rates than men (Figure 4). Over the last five match cycles (2019-2024), the average match rate for female applicants was 81.6% (n = 671/822) compared favorably to 70.4% (n = 1137/1614) for male applicants (p = 0.049). In 2024, 85% (n = 173/203) of female applicants matched versus 72% (n = 209/290) of male applicants​.

Racial/ethnic diversity (2024 data)

As noted, until recently, data on applicant race and ethnicity were not publicly reported by the AUA match. In 2024, the AUA provided a snapshot of the racial/ethnic breakdown of applicants and matched outcomes, allowing an initial look at the diversity in the urology pipeline. Among the applicants who identified a primary ethnicity in 2024 (n = 500), 46.8% were White (n = 234), 25.0% Asian (n = 125), 12.4% Hispanic/Latino (n = 62), 8.6% Black/African American (n = 43), 0.8% Native American/Alaska Native (n = 3), 0.2% Native Hawaiian/Pacific Islander (n = 1), and 6.4% other or undisclosed (n = 32). Match rates were highest for White and Asian candidates and lower for those from historically underrepresented groups. Specifically, approximately 81.6% of white applicants and 77.6% of Asian applicants matched (191 and 97 matched, respectively)​. In contrast, an estimated 69.8% of Black or African American applicants matched (n = 30/43) and about 65% of Hispanic/Latino applicants matched (n = 40/62).

## Discussion

In this 10-year analysis of urology residency match data, we observed notable growth in both the number of applicants and the available residency positions. The expansion of residency slots by approximately one-third reflects a positive response to rising concerns regarding workforce shortages. However, this increase has been met with a nearly proportional increase in applicants, maintaining the competitive nature of the urology match. With an average match rate of approximately 75%, urology remains one of the most competitive specialties. Each year, over 100 applicants do not match, representing not only personal setbacks but also a potential loss of qualified candidates to the field. Nonetheless, the >99% fill rate underscores the fact that programs consistently attract sufficient applicants, with almost no positions left unfilled.

Our findings reveal parallel growth in training capacity and interest in specialties. On average, 11 new residency positions are added annually. This expansion aligns with concerns from the AUA and other professional organizations that a significant proportion of the current workforce, nearly 30% of practicing urologists, is over the age of 65 [10]. Additionally, geographic maldistribution remains a challenge, as more than 60% of U.S. counties lack a practicing urologist [11]. While increasing residency positions helps accommodate more trainees, demand continues to outpace supply. From a policy perspective, a sustained investment in training infrastructure is warranted. Federal workforce projections, such as those from the Health Resources and Services Administration, consistently identify urology as a specialty at risk for future shortages [12]. Encouragingly, our data suggest that expansions in training slots, as seen in 2019 and 2020, correlate with improved match rates. This finding supports the notion that careful quality-preserving growth in residency programs may help alleviate workforce imbalances.

Consistent with previous findings, first-time senior U.S. applicants had the highest match rates, significantly outperforming previous graduates and international medical graduates (IMGs). This advantage is well documented across specialties and reflects systemic factors such as recent clinical experience, institutional support, and familiarity with the U.S. healthcare system [13-15]. While reapplicants may bolster their credentials before re-entering the match, they continue to face an uphill battle. Our findings suggest a modest upward trend in reapplicant success; however, applicants who go unmatched initially should be counseled on the challenges of reapplying. Similarly, IMGs face persistent barriers, with only approximately one-third matching. To improve outcomes, targeted guidance for these groups may include strategies such as obtaining U.S.-based research fellowships, securing strong letters of recommendation, and expanding the range of programs to which they apply.

Sex diversity in urology has shown encouraging progress. Over the past decade, the proportion of female residents has steadily increased, now comprising 30-40% of matched applicants in recent years. This mirrors broader shifts across medicine, where traditionally male-dominated fields are becoming more balanced [16]. A more gender-diverse urology workforce is not only a matter of equity but is also associated with better patient care, especially for diverse populations [17]. Interestingly, in recent cycles, women have matched at slightly higher rates than men. This trend may reflect the impact of deliberate efforts to support women in the field, such as mentorship initiatives, networking opportunities through the Society of Women in Urology, and institutional efforts to foster inclusive training environments.

Despite these advances, challenges in representation persist at the academic and leadership levels. Women currently hold only 16% of academic urology positions, and only 3% of department chairs and 8% of program directors are women [10]. While an increasing number of female residents offer hope for progress, systemic efforts are needed to ensure that this translates into leadership roles and long-term retention. Programs should proactively develop mentorship pathways, leadership training, and policies that support work-life balance and advancement opportunities for women.

Our analysis of racial and ethnic diversity, although limited to the 2024 cycle due to data availability, highlights persistent disparities. In 2024, Black and Hispanic applicants were significantly less likely to match than White and Asian applicants. The causes of this disparity are likely multifactorial, including differences in access to mentorship, geographic preferences, medical school resources, and potential bias (conscious or unconscious) within application and interview processes. Regardless of the cause, the underrepresentation of historically excluded groups remains a pressing concern. A more racially and ethnically diverse physician workforce improves access to care for underserved populations and enhances patient outcomes [17].

This study had several limitations. First, our data were obtained from publicly available aggregate-level reports. As such, we lacked individual-level data on applicant characteristics, such as exam scores, research output, geographic preferences, and interview invites, all factors that influence match outcomes. Second, data on race and ethnicity were available for only one year, limiting our ability to analyze trends over time. It is possible that 2024 may not fully represent the broader trends. Third, grouping applicants, such as combining U.S. MD and DO seniors or all international graduates, may have masked important subgroup differences. For example, U.S. citizen IMGs may have different outcomes from non-citizen IMGs. Finally, as an observational study, we can identify associations but cannot infer causality. Trends, such as rising female match rates or persistent disparities in underrepresented in medicine (URiM) outcomes, may be influenced by a range of unmeasured factors, including changes in specialty competitiveness, medical school class sizes, or external policy shifts.

Despite these limitations, our analysis offers a comprehensive overview of recent trends in urology residency matching and provides a foundation for future research. Further work is needed to examine the effectiveness of specific interventions, such as the role of diversity-focused mentorship programs or the impact of new funding on residency expansion. Additionally, it would be valuable to assess outcomes for unmatched applicants, whether they successfully match in subsequent years or enter other specialties. From a policy standpoint, addressing the imbalance between supply and demand in the urology workforce may require further increases in residency funding and strategies to incentivize practices in underserved areas.

## Conclusions

Over the past decade, urology matches have grown in both applicants and positions, maintaining a highly competitive environment. While match rates remain stable, disparities persist, particularly among reapplicants, international graduates, women in leadership, and underrepresented minorities. Continued expansion of training spots, targeted support for disadvantaged groups, and inclusive recruitment strategies are essential for addressing workforce shortages and improving equity within the field.
